# Detection of Temporal Changes in Insect Body Reflectance in Response to Killing Agents

**DOI:** 10.1371/journal.pone.0124866

**Published:** 2015-04-29

**Authors:** Christian Nansen, Leandro Prado Ribeiro, Ian Dadour, John Dale Roberts

**Affiliations:** 1 Department of Entomology and Nematology, University of California Davis, Davis, California, United States of America; 2 Department of Entomology and Acarology, University of São Paulo, Piracicaba, São Paulo, Brazil; 3 Centre for Forensic Science, The University of Western Australia, Perth, Western Australia, Australia; 4 School of Animal Biology and Centre for Evolutionary Biology and Centre of Excellence in Natural Resource Management, The University of Western Australia, Albany, Western Australia, Australia; Federal University of Viçosa, BRAZIL

## Abstract

Computer vision and reflectance-based analyses are becoming increasingly important methods to quantify and characterize phenotypic responses by whole organisms to environmental factors. Here, we present the first study of how a non-destructive and completely non-invasive method, body reflectance profiling, can be used to detect and time stress responses in adult beetles. Based on high-resolution hyperspectral imaging, we acquired time series of average reflectance profiles (70 spectral bands from 434-876 nm) from adults in two beetle species, maize weevils (*Sitophilus zeamais*) and larger black flour beetles (*Cynaus angustus*). For each species, we acquired reflectance data from untreated controls and from individuals exposed continuously to killing agents (an insecticidal plant extract applied to maize kernels or entomopathogenic nematodes applied to soil applied at levels leading to ≈100% mortality). In maize weevils (exposed to hexanic plant extract), there was no significant effect of the on reflectance profiles acquired from adult beetles after 0 and 12 hours of exposure, but a significant treatment response in spectral bands from 434 to 550 nm was detected after 36 to 144 hours of exposure. In larger black flour beetles, there was no significant effect of exposure to entomopathogenic nematodes after 0 to 26 hours of exposure, but a significant response in spectral bands from 434-480 nm was detected after 45 and 69 hours of exposure. Spectral bands were used to develop reflectance-based classification models for each species, and independent validation of classification algorithms showed sensitivity (ability to positively detect terminal stress in beetles) and specificity (ability to positively detect healthy beetles) of about 90%. Significant changes in body reflectance occurred at exposure times, which coincided with published exposure times and known physiological responses to each killing agent. The results from this study underscore the potential of hyperspectral imaging as an approach to non-destructively and non-invasively quantify stress detection in insects and other animals.

## Introduction

There has been a recent surge in studies, in which phenotypic responses by whole organisms are being quantified and carefully correlated with both molecular data and with performance or fitness under different environmental conditions. Mietchen et al. [1] demonstrated how magnetic resonance imaging and spectroscopy could be used as a non-invasive cryobiological tool to outline the distribution of water and crystals inside insect larvae down to the cellular level as part of studies into cold-hardiness of animals. Webster et al. [2] used near-infrared spectroscopy to successfully differentiate mated and unmated honey bee queens based on differences in reflectance profiles acquired from the bee abdomen. A critically important aspect of characterizing and quantifying phenotypic responses by whole organisms is the use of non-destructive and completely non-invasive imaging technologies, when data is acquired from the same individuals at multiple time points before and after exposure to controlled treatments. As an example, Nansen et al. [3] acquired reflectance data from three species of Australian tree seeds after different exposure times to conditions known to kill the seeds. The authors demonstrated that seed germination could be predicted with over 85% accuracy based on hyperspectral imaging data. In another study, Nansen et al. [4] acquired time series of high-resolution reflectance profiles from individual moth eggs, which had been parasitized by one of three species of minute juvenile egg-parasitoids (*Trichogramma*). Within 2–6 days after parasitism, the authors were able to accurately distinguish the host eggs with different *Trichogramma* instars based on species-specific changes in moth egg reflectance. Finally, Aw et al. [5] found that near-infrared spectroscopy of two species of fruit flies (*Drosophila melanogaster* and *D*. *simulans*) could be used to (classification accuracies ranging from 62% to above 90%) assess their gender, age them into two age-classes, and also to determine whether they were infected or not infected with *Wolbachia*.

The studies listed above underscore the important point, that internal (histological, physiological and biochemical) features of insects and other organisms may be detected and quantified non-invasively on the basis of features in reflectance profiles. Internal body features may be detected and characterized based on external body reflectance profiles, because of the “penetration depth” of the radiometric energy at different wavelengths [6]. For instance, a specific study of penetration depth into fruits showed that penetration depths reached 7.1 mm at 535 nm in plums and 13.8 mm at 720 nm for zucchini [7]. Aspects associated with penetration depth imply that reflectance profiles acquired from either plant tissues or insect bodies are provide information about the physiological and biochemical composition of internal tissues.

The insect cuticle plays many critically important physiological and ecological roles, including [8]: thermo-regulation, intra-specific communication, camouflage/mimicry, water management, and defence against pathogens. Furthermore, there is ample evidence of the insect cuticle being highly dynamic with its chemical composition responding to age of individuals [9–13] and a wide range of behavioural [9,14–17] and environmental factors [9, 14]. Melanin is responsible for brown or black colouring of insects, [18–20], and the biochemical precursors leading to melanin synthesis also trigger a range of immune responses [21,22]. Thus, there may be multiple and complex interactions between internal physiological processes and the biochemical composition of the cuticle. The latter supports the hypothesis, that the biochemical composition (and therefore also body reflectance profiles) vary between stressed and non-stressed insect individuals.

In this study, we addressed the questions: 1) whether exposure to known killing agents would cause a detectable change in body reflectance? 2) And if so, after what exposure time? We acquired time series of hyperspectral imaging data from: 1) Adult maize weevils (*Sitophilus zeamais* Motsch) (Curculionidae: Coleoptera) in Petri dishes containing maize kernels (*Zea mays* L) with/without a hexanic plant extract, which is known to kill these beetles [23] and cause acetogenins action (mainly muscle coordination loss) in maize weevils after 12–20 hours of exposure [24]. 2) Adult larger black flour beetles (*Cynaeus angustus* LeConte) (Tenebrionidae: Coleoptera) in Petri dishes containing soil with/without entomopathogenic nematodes (*Steinernema carpocapsae* Weiser), which are known to kill these beetles within 24–48 hours [25]. As we only used mature adult insects, we assumed that: 1) the reflectance profiles acquired from control beetles should remain constant over time, 2) the reflectance profiles acquired from control beetles and beetles exposed to a killing agent (hexanic plant extract or entomopathogenic nematodes) were similar at the onset of the study, and 3) that reflectance from beetles exposed to a killing agent would diverge over time and therefore indicate terminal stress. Our prediction of reflectance-based detection of stress in adult beetles is a logical development of the use of hyperspectral imaging technology to classify objects with/without stress—including growing plant leaves [26,27], seeds [3,28,29], and insect eggs containing developing parasitoids [4].

## Materials and Methods

We chose mature adult beetles with fully sclerotized exoskeletons for this study, as we assumed these individuals to have similar and consistent reflectance profiles during the course of the study. Larvae of each species would clearly have been more susceptible to both killing agents, but they grow and moult and are therefore expected to change considerably in terms of reflectance profiles, even within a few days. With lower susceptibility to killing agents and more stable reflectance profiles, adult life stages were considered more appropriate for detection of stress induced by the killing agents.

### DATA SET 1: MAIZE WEEVILS

A continuous colony of maize weevils is maintained on maize kernels and reared under controlled conditions (20–22°C and 50–70% RH) at the Department of Entomology and Acarology, University of São Paulo. Maize weevils can be readily reared on whole maize kernels, and in this study they were raised on hybrid AG 1051 maize kernels (yellow-toothed, semi-hard), which had been conditioned to a moisture content of 12.5% (by weight). No specific permissions were required for rearing of these beetles under university laboratory conditions. Groups of 10 unsexed mature adult maize weevils were transferred to 9 cm diameter Petri dishes containing 10 g of maize kernels with/without treatment with a slow-acting killing agent and we characterized the change in weevil reflectance over time. We used 10 replicates for each treatment. Experimental maize kernels were treated with a hexanic extract from *Annona mucosa* Jacq. (Annonaceae) seeds. The hexanic plant extract was prepared by cold plant maceration in hexane solvent as described in [23]. This hexanic extract inhibits complex I in the mitochondrial respiratory chain of target arthropods [30]. Insects exposed to hexanic extracts from *A*. *mucosa* seeds are characterized by inactivity, instability and loss of muscle coordination, paralysis and slow but inevitable death due to respiratory failure. Based on a previous study [23], maize kernels were treated with 259.31 ppm of this hexanic plant extract, as it kills 90% of maize weevils within 10 days of exposure allowing. Thus, this concentration allowed us to study the temporal progress of terminal stress. The hexanic plant extract was applied with a microatomizer coupled to a pneumatic pump and adjusted to deliver a spray volume of 30 ml per kg grain [23]. We also included maize kernels treated with hexane only as a control treatment. Hyperspectral images of beetles in individual Petri dishes were acquired after: 0, 12, 36, 60, and 144 hours of exposure. This data set comprised a total of 100 hyperspectral images (2 treatments × 5 exposure times × 10 replicates).

### DATA SET 2: LARGER BLACK FLOUR BEETLES

Colonies of larger black flour beetles were reared in jars with soil and potato slices and maintained under controlled conditions at the Texas AgriLife Research Center, Lubbock, TX, at 30°C and 35–40% relative humidity [25]. No specific permissions were required for rearing of these beetles under university laboratory conditions. A culture of the entomopathogenic nematode, *S*. *carpocapsae* (strain NY 001), was maintained by continuous infection of greater wax moth larvae (*Galleria mellonella* F. (Lepidoptera: Pyralidae) according to [25]. We prepared two 1 kg batches of soil with a 10% (by weight) moisture content, and the equivalent of about 1,000 infective *S*. *carpocapsae* juveniles were thoroughly mixed into 50 g of soil in each of five 9 cm diameter Petri dishes, while an additional five Petri dishes were used as untreated controls. Groups of five unsexed larger black flour beetles were transferred to Petri dishes containing soil with, or, without entomopathogenic nematodes. Hyperspectral images were acquired from each Petri dish after: 0, 4, 9, 21, 26, 45, and 69 hours of exposure to entomopathogenic nematodes. This data set comprised a total of 70 hyperspectral images (2 treatments × 7 exposure times × 5 replicates).

### HYPERSPECTRAL IMAGING

**Fig 1:**
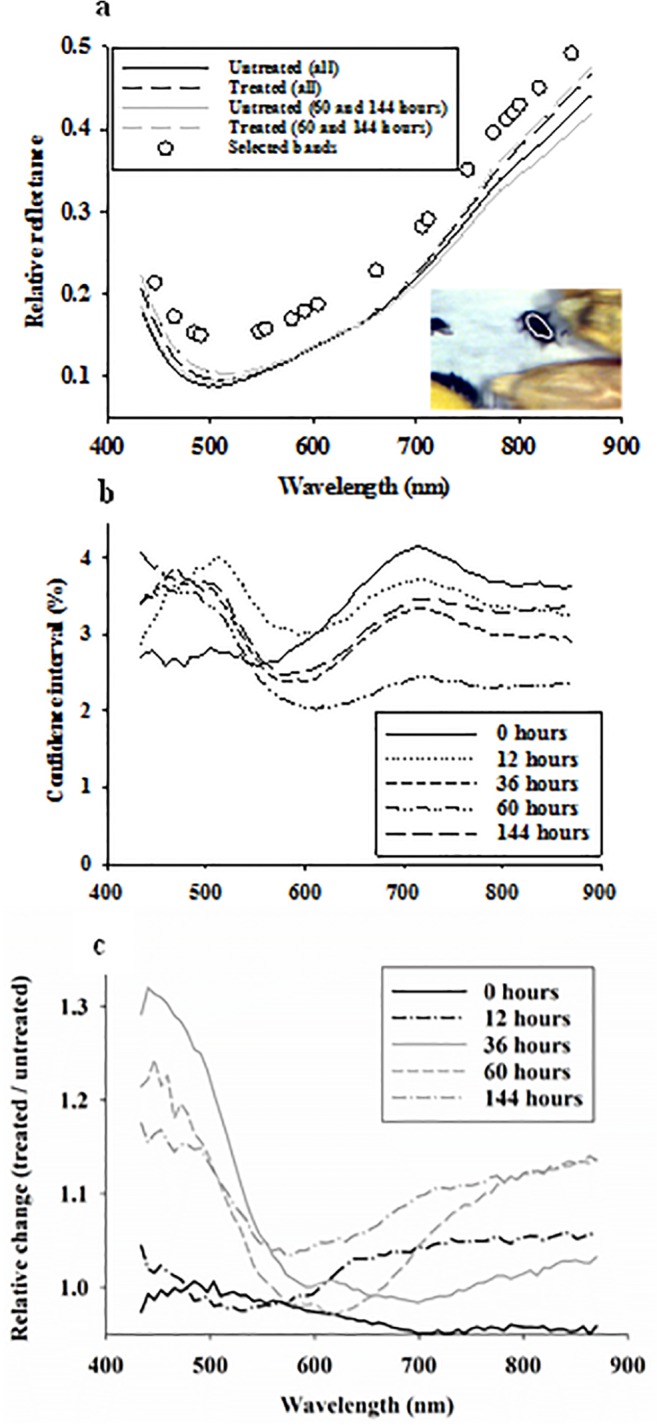
Average body reflectance profiles acquired from maize weevils on maize kernels with/without hexanic plant extract across all exposure times or for exposure times of 60 and 144 hours **(a)**. Open dots represent spectral bands selected in linear discriminant classification of healthy and terminally stressed weevils. 95% confidence intervals as percentage of average reflectance in each spectral band (**b**). Relative effect (treated / untreated) of hexanic plant extract treatment on body reflectance profiles from maize weevils for all five exposure times (0–144 hours) (**c**).

**Fig 2:**
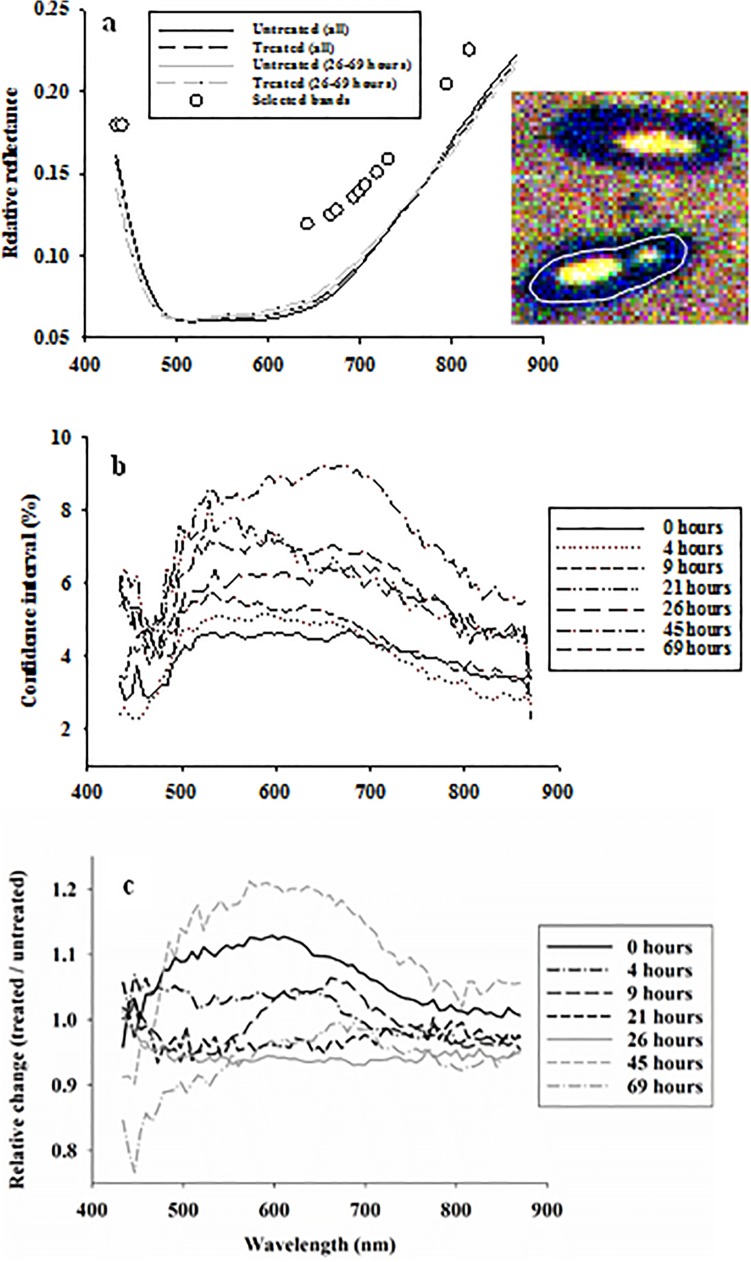
Average body reflectance profiles acquired from larger black flour beetles exposed to soil with/without entomopathogenic nematodes **(a)**. Open dots represent spectral bands selected in linear discriminant classification of healthy and terminally stressed weevils. 95% confidence intervals as percentage of average reflectance in each spectral band (**b**). Relative effect (treated / untreated) of hexanic plant extract treatment on body reflectance profiles from larger black flour beetles for all seven exposure times (0–69 hours) (**c**).

Hyperspectral imaging refers to acquisition of reflectance data with a high spectral resolution (typically >100 narrow spectral bands) with each pixel in an image being associated with a detailed reflectance profile. A standard spectrometer averages the reflectance over a certain area, so it collects the average reflectance from “a single pixel image”. One of the advantages of acquiring hyperspectral imaging data is that pixels can be selected carefully to only represent the desired target object and reflectance data from background and undesired features within the image can be omitted either manually [31] or through applications of radiometric filters [3,4,32,33]. After hyperspectral image acquisition, we inspected each hyperspectral image and carefully selected and averaged 20–200 pixels from the dorsal side of individual beetles (see images in Figs 1A and 2A). Similar to previously published studies [4,29], input data were acquired at a spatial resolution of about 45 pixels (reflectance profiles) per mm^2^. Thus, selection of 20–200 pixels from the dorsal side of individual beetles could be done without concerns about the signal/noise ratio, as pixels representing background could easily be avoided. Due to movement of individuals during imaging and because the hyperspectral camera is a line-scanning device, some of the acquired profiles from beetles represented only parts of their bodies.

We used a hyperspectral push broom spectral camera (PIKA II, Resonon Inc., Bozeman, MT), which collects 160 bands with a spectral resolution of 3.1 nm in the range from 405 to 907 nm. However, to avoid spectral bands with low signal/noise ratio, only 140 spectral bands from 434–876 nm were included. The objective lens had a 35 mm focal length (maximum aperture of F1.4) and was optimized for the visible and NIR spectra. The main specifications of the spectral camera are as follows: Firewire (IEEE 1394b) interface, 12 bit digital output, 7^o^ angular field of view. All hyperspectral images were collected with artificial lighting from 15 W, 12 V light bulbs mounted in 2 angled rows, one on either side of the lens, with 3 bulbs in each row. A voltage stabilizer (Tripp-Lite, PR-7b, www.radioreference.com) powered the lighting. Ambient climate conditions were between 19–22 °C and 30–40% relative humidity. A piece of white Teflon (K-Mac Plastics, MI, USA) was used for white calibration, and “relative reflectance” refers to proportional reflectance compared to that obtained from Teflon and from dark calibration. Consequently, relative reflectance values in each spectral band ranged from 0 to 1.

Due to concerns about model over-fitting [32, 33], we conducted spectral binning by averaging the 140 spectral bands from 434–876 nm into 70 spectral bands with a spectral range of 6.2 nm. Generally, there is a high risk of over-fitting of classification models when the number of explanatory variables (i.e., spectral bands) is similar to or exceeds the number of samples (average reflectance profiles) being analysed. Model over-fitting may lead to low robustness/repeatability when the classification model is applied to independent validation data [34,35]. Through spectral binning into 70 spectral bands, the risk of model over-fitting was considered negligible. In addition, independent validation data were used to confirm the robustness of the classification models.

### ACQUISITION OF AVERAGE REFLECTANCE PROFILES FROM ADULT BEETLES

An important aspect of this study was to impose minimum disturbance to beetles in each Petri dish, as disturbance or stress might elicit a stress response, which could affect or bias the acquired reflectance data. Consequently, no or few attempts were made to influence the position of beetles within each Petri dish during acquisition of hyperspectral images. Individual beetles could either be hiding underneath maize kernels or partly buried in the soil. We repeated an image acquisition, if less than two beetles were imaged for a particular combination of treatment and exposure time. With the objective being to acquire average reflectance profiles from beetles, marking of individuals was not an option, so it was not possible to assess which individuals were imaged at each imaging event. We obtained the following numbers of average reflectance profiles from the two data sets: 1) 284 larger black flour beetles from 100 hyperspectral images, and 2) 241 maize weevils from 70 hyperspectral images.

### DATA ANALYSIS

**Fig 3:**
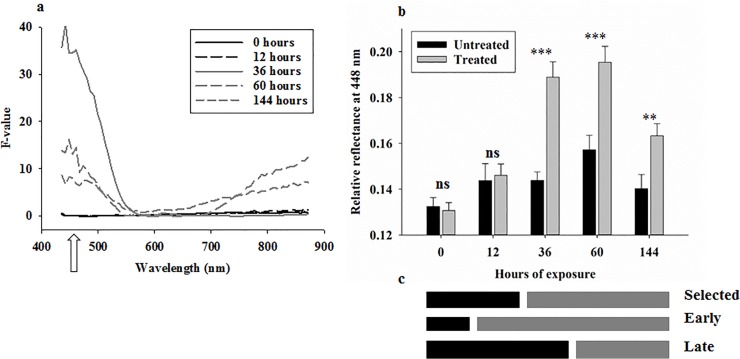
Results (F-values) from analyses of variance in 70 spectral bands from 434–876 nm of reflectance data from maize weevils on maize kernels with/without hexanic plant extract **(a)**. Separate analyses were conducted for all combinations of spectral bands and exposure times. Arrow indicates position of spectral band at 448 nm. Average reflectance at 448 nm from maize weevils exposed to maize kernels with/without hexanic plant extract (**b**). Selection of time intervals for treatment responses according to results in b (selected) or one time point earlier (early) or later (late) (**c**). “*” denotes difference at the 0.05-level, “**” denotes difference at the 0.01-level, and “***” denotes difference at the 0.001-level.

**Fig 4:**
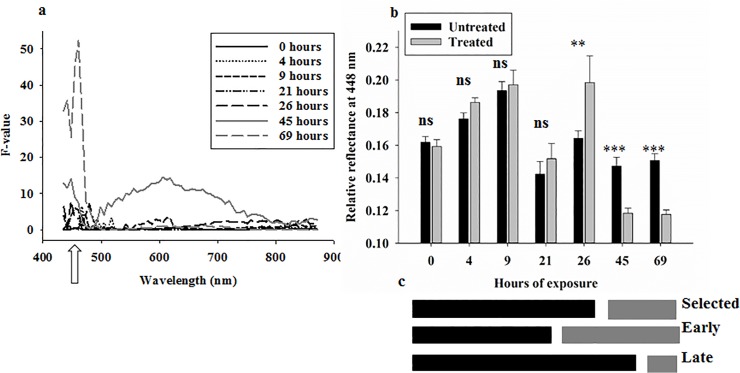
Results (F-values) from analyses of variance in 70 spectral bands from 434–876 nm of reflectance data from larger black flour beetles on soil with/without entomopathogenic nematodes **(a)**. Separate analyses were conducted for all combinations of spectral bands and exposure times. Arrow indicates position of spectral band at 448 nm. Average reflectance at 448 nm from larger black flour beetles exposed to soils with/without entomopathogenic nematodes (**b**). Selection of time intervals for treatment responses according to results in b (selected) or one time point earlier (early) or later (late) (**c**). “*” denotes difference at the 0.05-level, “**” denotes difference at the 0.01-level, and “***” denotes difference at the 0.001-level.

All data analysis was conducted in PC-SAS 9.3 (SAS Institute, NC). Initially, separate analyses of variance (PROC ANOVA) were conducted for each species and for combination of spectral band and time points. Regarding the data set acquired from maize weevils, a total of 350 analyses of variance were conducted (70 spectral bands × 5 time points), and 490 analyses of variance were conducted with the data set acquired from larger black flour beetles (70 spectral bands × 7 time points). In each combination of beetle species, time point, and spectral band, we compared average reflectance values from treated and untreated beetles. For each beetle species, we wanted to identify spectral bands with a significant response to treatment and also with a significant response over time. In both beetle species, this pattern of significant treatment response was identified in spectral bands between 434 to 550 nm. In maize weevils, indication of stress (significant change in body reflectance) was detected in spectral bands 12–36 hours after exposure, while in the larger, black flour beetles the perceived stress response was detected in reflectance profiles acquired 26–45 hours after exposure. Thus, these exposure times were “selected” as being the ones, in which a reflectance response to treatment was expected (Figs 3B and 4B). Subsequently, we dichotomised the reflectance data, so that all reflectance profiles acquired from untreated beetles and from treated beetles before the onset of the stress response were assigned a “0”, and reflectance profiles acquired from adult beetles in treated samples after the detection of a stress response were assigned a “1”. For each beetle species, we conducted two additional dichotomous divisions of the data, in which treatment effects were considered either one time point earlier (denoted “early) or later (denoted “late”) compared to the “selected” onset of treatment response.

The dichotomous dummy variable was used as response variable in linear discriminant analysis [36] of the three data sets for each species (“selected”, “early”, and “late”). We divided data set for each beetle species into three randomly selected thirds, and two-thirds of the data was used as training data to develop the linear discriminant classification, and the remaining one-third was used for independent validation. Each of the thirds of each data was used for independent validation, so three linear discriminant classifications were conducted for each data set. All linear discriminant classifications were assessed based on sensitivity (ability to positively detect terminal stress) and specificity (ability to positively detect untreated controls) of independent validation data. The same procedure was followed for all linear discriminant classifications. Initially, we used forward stepwise selection (PROC STEPDISC) to select (out of the 70 spectral bands) only the spectral bands with significant contributions to the classification model. Subsequently, we conducted linear discriminant analysis (PROC DISCRIM) with the selected spectral bands to generate a classification model (based on two-thirds of the data), Finally, the classification accuracy of the model was quantified based on independent validation (the remaining third of the data).

## Results

### REFLECTANCE RESPONSES BY MAIZE WEEVILS

Across all five time points, average reflectance profiles from adult maize weevils exposed to a hexanic plant extract (across all exposure times) were marginally higher, especially in spectral bands below 500 nm and above 750 nm, than average reflectance from control maize weevils (Fig 1A). Based on 95% confidence levels (as percentage of average), it is seen that reflectance values showed similar distribution range (varying between 2–4%) both over time and across the 70 spectral bands between 434–876 nm (Fig 1B). The treatment effect became more pronounced after dividing average reflectance profiles from stressed beetles with those from healthy beetles (Fig 1C). From this visualisation of relative treatment effect, it is seen that: 1) average reflectance profiles from healthy and stressed maize weevils were very similar (close to one) after 0 and 12 hours of exposure, and 2) there was a 15–35% increase in average reflectance in spectral bands from 434–500 nm after 36–144 hours of exposure. Fig 3A summarizes the results (F-values) from 350 analyses of variance, and there was no significant effect of the hexanic plant extract on reflectance profiles acquired from adult beetles after 0 and 12 hours of exposure. After 36 hours of exposure, there was a highly significant treatment response in all spectral bands from 434–550 nm, but the remaining spectral bands showed no significant treatment response. After 60 or 144 hours of exposure, there was still a significant treatment response in spectral bands from 434–550 nm, and spectral bands from 800–872 nm also showed a significant treatment response. Thus, the 350 analyses of variance showed that exposure to the hexanic extract had no significant effect on body reflectance in most of the spectral bands, but one particular portion of the blue-green region of the radiometric spectrum (434–550 nm) responded significantly at all time points 36–144 hours after exposure.

As an illustration of the reflectance response in the blue-green region of the radiometric spectrum, average reflectance in a single spectral band at 448 nm showed a strong response to hexanic plant extract treatment, and from pairwise treatment comparisons we found that hexanic plant extract (Fig 3B): 1) caused no significant increase in reflectance within 0–12 hours of exposure, and 2) caused a highly significant increase in reflectance after 36–144 hours of exposure.

The 350 analyses of variance could be considered an exploratory approach to identify at what time point the treatment effect was expressed in terms of body reflectance, and 36 hours of exposure was selected as the time point with a significant reflectance response to the killing agent (Fig 3C). Two additional time points, one observation earlier or later, were also examined. Using this dummy variable as response in a stepwise linear discriminant analysis, 19 spectral bands were found to contribute significantly to the classification of adult maize weevils with or without exposure to the hexanic plant extract. The selected spectral bands were located across the entire spectral range (Fig 1A). Table 1 summarizes the average classification results from the three validations of linear discriminant analyses with sensitivity (ability to positively detect terminal stress in adult beetles) of about 89% and specificity (ability to positively detect healthy beetles) of about 94%. It is not surprising that specificity was slightly higher than the sensitivity, as it is possible that some of the beetles exposed to the hexanic plant extract were not stressed. However, none of the beetles from untreated maize were expected to show signs of stress induced by the killing agent. For comparison, classification results from “early” (assuming treatment effect after 12–144 hours of exposure) (sensitivity = 78% and specificity = 87%) and “late” (assuming treatment effect after 60–144 hours of exposure) (sensitivity = 84% and specificity = 86%) responses to treatment were lower than obtained with 36 hours of exposure as the selected time point for stress detection.

**Table 1:** Average classification accuracy (%) of beetles with/without exposure to terminal stress.

Maize weevils		
	Treated (observed)	Control (observed)
Treated (actual)	91.04	8.96
Control (actual)	11.12	88.88
	Sensitivity	Specificity
	89.11	90.84
Larger black flour beetles	
	Treated (observed)	Control (observed)
Treated (actual)	93.09	6.91
Control (actual)	7.41	92.59
	Sensitivity	Specificity
	92.63	93.06

“Sensitivity” denotes the ability to accurately detect/classify terminal stress, and “specificity” denotes the ability to accurately detect/classify untreated controls.

### REFLECTANCE RESPONSES BY LARGER BLACK FLOUR BEETLES

Average reflectance from adult larger black flour beetles exposed to entomopathogenic nematodes (across all exposure times) was only marginally higher compared to average reflectance from untreated controls (Fig 2A). The confidence intervals showed similar spectral patterns over time, with highest variance in spectral bands from 500–700 nm, but the amplitude of variance varied inconsistently over time within 3%-9% of average reflectance values (Fig 2B). Dividing average reflectance profiles from stressed adult beetles with those from healthy beetles, it was seen that exposure to the killing agent caused a considerable decrease in reflectance in spectral bands from 434–480 nm after 45–69 hours of exposure (Fig 2C). Fig 4A summarizes the results from 490 analyses of variance, and the strongest and consistently significant response was seen in spectral bands from 434–480 nm after 45 and 69 hours of exposure. Several of the bands in this spectral range also responded significantly after 26 hours of exposure. After 45 hours of exposure, there was a significant treatment response in spectral bands from 500–700 nm (responsible for the higher average reflectance in Fig 2C), but this response was not observed after longer exposure to the killing agent and could therefore not be considered a reliable indication of stress.

Average reflectance in a single spectral band at 448 nm was selected based on the strong response to nematode treatment, and a highly significant decrease in reflectance after 45–69 hours of exposure was observed (Fig 4B). As in the study of body reflectance from maize weevils, a dummy variable was selected, in which “1” denoted exposure to nematodes for 45 or 69 hours, and “0” was assigned to average reflectance profiles acquired from adult beetles of all other combinations of treatment and exposure time (Fig 4C). Stepwise linear discriminant analysis selected 13 spectral bands, which were predominantly located in the spectral range 630–720 nm (Fig 2A). The average classification results (in %) from the three validations of linear discriminant analyses showed sensitivity of about 92% and specificity of about 93% (Table 1). Thus, we demonstrated that time series reflectance data (hyperspectral imaging) acquired from both beetle species could be used to detect stress with high accuracy (high sensitivity) and with low level of false positives (high specificity). For comparison, classification results from “early” (assuming treatment effect after 21–69 hours of exposure) (sensitivity = 74% and specificity = 90%) and “late” (assuming treatment effect after 45–69 hours of exposure) (sensitivity = 100% and specificity = 91%) responses to treatment were generally lower than obtained with 45 hours of exposure as the selected time point for stress detection.

## Discussion

Due to the penetration depth of the radiometric energy in spectral bands from 400–900 nm being several mm [7,37], it seems reasonable to assume that hyperspectral imaging of external insect bodies, as used in this study, can be deployed non-destructively to living insects to detect internal physiological and biochemical responses to treatments (including stress response to killing agents). The results from both beetle studies supported all three assumptions concerning selected spectral bands, as it was possible to select portions of the examined wavelength spectrum, in which body reflectance: 1) from untreated control beetles did no vary significantly over time, 2) from untreated control beetles did no vary significantly over time at the onset of the study, and 3) from beetles exposed to a killing agent diverged after a certain exposure time. Consequently, the results appear to support the hypothesis that non-destructive acquisition of body reflectance profiles of insects can be used to assess their health status.

Death, or stress ultimately leading to death, is undoubtedly one of the most significant events in an organism’s life, and detection and characterization of “terminal stress” as an irreversible process leading to death is therefore critically important. In humans, death represents permanent cessation of all biological functions that sustain a particular living organism, including loss of: pulse, breathing, reflexes, responses to normally painful stimuli, signal on electroencephalogram, and blood flow to the brain [38]. However diagnostic tools to detect and characterize death or “terminal stress” in animals have received much less attention. Furthermore, in many small animals with very sessile life strategies and life stages, it can be quite challenging to determine whether an individual is dead or not. Due to their small body size, insertion of instruments into tissues or collection of gas exchange measurements become challenging. In addition, many animals perform tonic immobility or thanatosis, so mobility and other observational criteria may be less conclusive.

There is a wealth of studies describing how reflectance profiles of plants are affected by a wide range of abiotic and biotic factors [26,39,40], and of how food items with/without internal insect damage are distinguishable based on reflectance profiling [29,32,41–44]. However, none of these studies acknowledge or demonstrate, that before imposing stress or damage the imaged organisms in the different treatment groups were similar/identical in terms of reflectance. That is, rarely are time series of reflectance data acquired and analysed to demonstrate a shift in reflectance in response to a particular treatment. In a typical reflectance-based study, plants may have been grown under stress regimes (abiotic deficiencies or exposed to a herbivore), but what combination of stress intensity and exposure time is needed to create a significant change in reflectance profiles?

Regarding the exposure time points, at which a significant change in body reflectance was detected is supported by known effects of both killing agents. The hexanic plant extract inhibits complex I in the mitochondrial respiratory chain of target arthropods [30]. Using the same concentration of the hexanic plant extract as used in this study, Ribeiro [24] showed that symptoms of acetogenins action (mainly muscle coordination loss) in maize weevils started to occur after 12–20 hours of exposure. Thus, there appears to be a strong correlation between acetogenins action induced by the hexanic plant extract and the significant change in body reflectance. After infective juveniles have successfully entered the insect host, the entomopathogenic nematodes (*S*. *carpocapsae*) propagate and secrete toxins (produced by a bacterium in the genus *Xenorhabdus*), and insect death typically occurs within 48–72 h of parasitism [45]. Several studies have demonstrated that only a few infective juveniles are required to kill various insects, and Rosa et al. [45] monitored mortality of sixth-instar moth larvae of *Pseudaletia unipuncta* over time and described its association with build-up of *S*. *carpocapsae* infective juveniles inside the host. Based on studies involving exposure time and dosages of and *S*. *carpocapsae* infective juveniles, the authors concluded that 60–100 hours would cause 50–100% mortality of the host. The results by Rosa et al. [45] are not directly comparable to the ones from the current study, as insect host species and life stages vary in susceptibility to *S*. *carpocapsae* and other entomopathogenic nematodes. Nansen et al. [25] showed that larger black flour beetles exposed to *S*. *carpocapsae* were dead within 24–48 hours of exposure time. It is therefore quite noteworthy that a significant change in body reflectance was detected within that time period.

There is ample evidence of positive relationships between body tissue content of fluorescent age pigments, lipofuscins, and age of individuals [46,47]. In addition, numerous studies have described how epicuticular composition of insects varies: 1) among life stages and age of adults [9–13], 2) among eusocial individuals with different tasks [16,17], 3) among conspecific individuals with different mating status [9,14], 4) among conspecific individuals reared on different diets [9, 14], and 5) in response to physiological changes induced by conspecific density [15]. Thus, there is growing evidence of how reflectance profiling can be used non-destructively to identify and characterize physiological responses to environmental conditions. Each application of imaging technologies will likely require a specific study to identify the combination of spectral bands with the strongest and most consistent response to treatment factors. Moreover, if the reflectance response by an organism is dependent on which stressor is being evaluated, then it may be possible to distinguish between stressors and possibly group them into categories based on reflectance responses.

In “phenomics”, phenotypic responses are being quantified and carefully correlated with both molecular data and with performance or fitness under different environmental conditions [48,49]. Large plant phenomics facilities are being developed, such as the Australian Plant Phenomics Facility (http://www.plantphenomics.org.au/) and the National Plant Phenomics Centre in the UK (http://www.plant-phenomics.ac.uk/en/). The establishment of such research centres emphasizes the growing interest in phenotypic response by whole organisms to a wide range of abiotic and biotic conditions and stressors. We argue that integration of hyperspectral imaging technologies and classification of features in reflectance profiles into phenomics studies of both plants and animals will be a major research frontier in the coming decades. Based on independent validation of classification algorithms, we showed sensitivity (ability to positively detect terminal stress in beetles) and specificity (ability to positively detect healthy beetles) were about 90–92% in both species. Such high classification accuracies are noteworthy when considering that: 1) body reflectance profiles were acquired from adult beetles with a fully sclerotized exoskeleton, and 2) no attempts were made to place beetles in specific positions to account for spectral noise imposed by variation in projection angles and differences in reflectance from different parts of the insect body. We are not aware of any published studies describing non-destructive and non-invasive methods to detect stress in arthropods. Furthermore, we argue that the method presented is of relevance to a wide range of researchers conducting molecular, physiological, behavioural, and evolutionary research studies involving responses by animals to biological and environmental factors.
